# No evidence of BoHV-1 exposure and low levels of *pestivirus* exposure in sera from 116 opportunistically sampled wild deer in Northern Ireland

**DOI:** 10.1186/s13620-025-00292-5

**Published:** 2025-10-28

**Authors:** Maggie Lyons, Angela Lahuerta-Marin, Joe Clarke, Asa Moyce, James McConville, Siobhan Porter, Maria Guelbenzu-Gonzalo, Ronan O’Neill, Sharon Verner, Eric R. Morgan

**Affiliations:** 1 Queen’s University Belfast, School of Biological Sciences, 19 Chlorine Gardens, Belfast, BT9 5DL Ireland; 2 Agri-Food and Biological Institute Northern Ireland, 12 Stoney Road, Stormont, Belfast, BT4 3SD Ireland; 3 Animal Health Ireland, 2–5 The Archways, Co. Leitrim, Carrick On Shannon, N41 WN27 Ireland; 4 Virology Division DAFM Laboratories Backweston, Co. Kildare, Celbridge, W23 VW2C Ireland; 5 Animal Health and Welfare Northern Ireland, Unit 49, Dungannon Enterprise Centre, 2 Coalisland Rd, Dungannon, Co. Tyrone BT71 6JT Ireland

**Keywords:** Deer, Wildlife, Bovine Viral Diarrhoea (BVD), Border Disease (BD), Infectious Bovine Rhinotracheitis (IBR), Pestivirus, Northern Ireland

## Abstract

Bovine Viral Diarrhoea Virus (BVDV), Border Disease Virus (BDV), and Bovine Herpesvirus-1 (BoHV-1, the cause of Infectious Bovine Rhinotracheitis, IBR), are economically important endemic viruses in ruminant livestock in the United Kingdom and Ireland. Deer could undermine control efforts in livestock by contributing to virus transmission and maintenance, but information on the presence of these viruses in the wild deer population is lacking. Blood samples from wild fallow and sika deer culled in Northern Ireland were collected opportunistically in the 2022–23 hunting season and tested using enzyme-linked immunosorbent assay (ELISA) for the presence of antibodies to these viruses (*n* = 116). No antibodies against BoHV-1 were detected. Antibodies against *pestivirus* were detected in three samples (2.6%), all from sika deer, and constitute the first report in this species in Europe. Virus strain differentiation by virus neutralization test (VNT) was inconclusive. Results therefore indicate no evidence of exposure to BoHV-1 and very low levels of *pestivirus* exposure in these deer populations. Based on these results there are currently no grounds to implicate deer as significant wildlife reservoirs of these viruses.

## Background

Diseases caused by viruses are among the most significant economic and welfare drains on the livestock industry, including in cattle [1–3]. Bovine Viral Diarrhoea (BVD), for instance, is estimated to cost the cattle industry in the United Kingdom (UK) up to £552 per cow per year [4], and the Northern Irish industry £25–30 million per year [5]. The aetiological agent of BVD is the Bovine Viral Diarrhoea Virus (BVDV), an RNA *Pestivirus* of the genus Flaviviridae, with at least two viral species (*Pestiviruses A* and *B*, previously BVDV-1 and BVDV-2, respectively)*,* each with several genotypes [6–10]. Border Disease (BD), also known as Hairy Shaker Disease, is among five ‘iceberg diseases’ of sheep; i.e., slow onset, production limiting diseases for which clinical incidence underestimates true burden [10]. It is caused by *Pestivirus D* (previously BDV). *Pestivirus* transmission dynamics and pathogenesis are complex, with both horizontal and vertical routes of transmission, and BVDV and BDV infections can be transient or persistent [11–15]. *Pestiviruses* can also infect multiple artiodactyl species and are not restricted to their main domestic hosts. Thus, sheep have been reported to carry BVDV at higher rates than BDV [14, 16] and cattle are susceptible to BDV [15]. Similarly, deer are susceptible to *pestiviruses* of domestic ruminants [17–20]. The potential of deer to act as reservoirs for these viruses is a poorly understood biosecurity risk, especially against the backdrop of eradication and control efforts in livestock.

Infectious Bovine Rhinotracheitis (IBR) is caused by Bovine *alphaherpesvirus* −1 (BoHV-1), a virulent subtype of which (BoHV-1.1) emerged in the USA in the 1950s [21] and subsequently spread to and throughout Europe in the 1970s [22]. The disease in cattle presents as an acute illness, following which the virus infiltrates local sensory neurons and eventually progresses to the trigeminal ganglia [23, 24], where it remains latent until stress-induced reactivation. Although morbidity and mortality rates in cattle are typically low, ~ 7.6% and 3.0% respectively [22], the disease is costly to the livestock industry, especially through its contribution to the multifactorial bovine respiratory disease complex [2, 3]. Gene conservation across alpha *herpesvirus* species has resulted in high antigenic homogeneity [24], which can lead to cross-reactivity on diagnostic testing and cross-species transmission. Bovines are susceptible to cervine, bubaline, caprine and elk strain *alphaherpesvirus* −1 (CvHV-1, BuHV-1, CpHV-1 and ElkHV-1, respectively) and conversely, each of those ruminants can be infected by bovine strain alphaherpesvirus-1 (BoHV-1) [25]. Despite their potential epidemiological importance, BoHV-1 infections in deer are understudied.

In the Republic of Ireland (ROI), a compulsory BVDV eradication scheme in cattle (co-ordinated by Animal Health Ireland, AHI) came into effect in 2013, and Northern Ireland followed in 2016 (co-ordinated by Animal Health and Welfare Northern Ireland, AHWNI). At the beginning of the ROI programme, figures showed 11.3% herd prevalence and 0.7% animal prevalence. In 2021 those figures had decreased to 0.3% and 0.01%, respectively, thus the ROI’s request for official freedom status is under consideration and expected to be granted in the very near future [26]. In Northern Ireland the cattle herd level prevalence as of January 2023, was 3.8% [27]. A 2020 study estimated Northern Irish sheep flock *pestivirus* prevalence at 17.6% (1.6% individual animal prevalence) [14]. As of 2024, the UK and ROI have yet to implement compulsory BoHV-1 (IBR) eradication policies, although AHI is awaiting roll-out of such a scheme in ROI [28].

Northern Ireland, like many other countries in which these viruses are endemic, has an increasing wild deer population [29, 30], and studies have shown that many deer species are susceptible to livestock *pestiviruses* and BoHV-1, both naturally [31–35] and experimentally [36–39]. Biosecurity measures adopted for virus control in livestock may, therefore, be undermined by the presence of infection in wild deer [40–43], and this is an important factor to consider when aiming for eradication. Here, an opportunistic survey was performed to determine whether wild deer in Northern Ireland are infected with BVDV, BDV and BoHV-1, and hence their importance to virus control plans for livestock.

## Methods

### Sampling Methodology

**Fig. 1:**
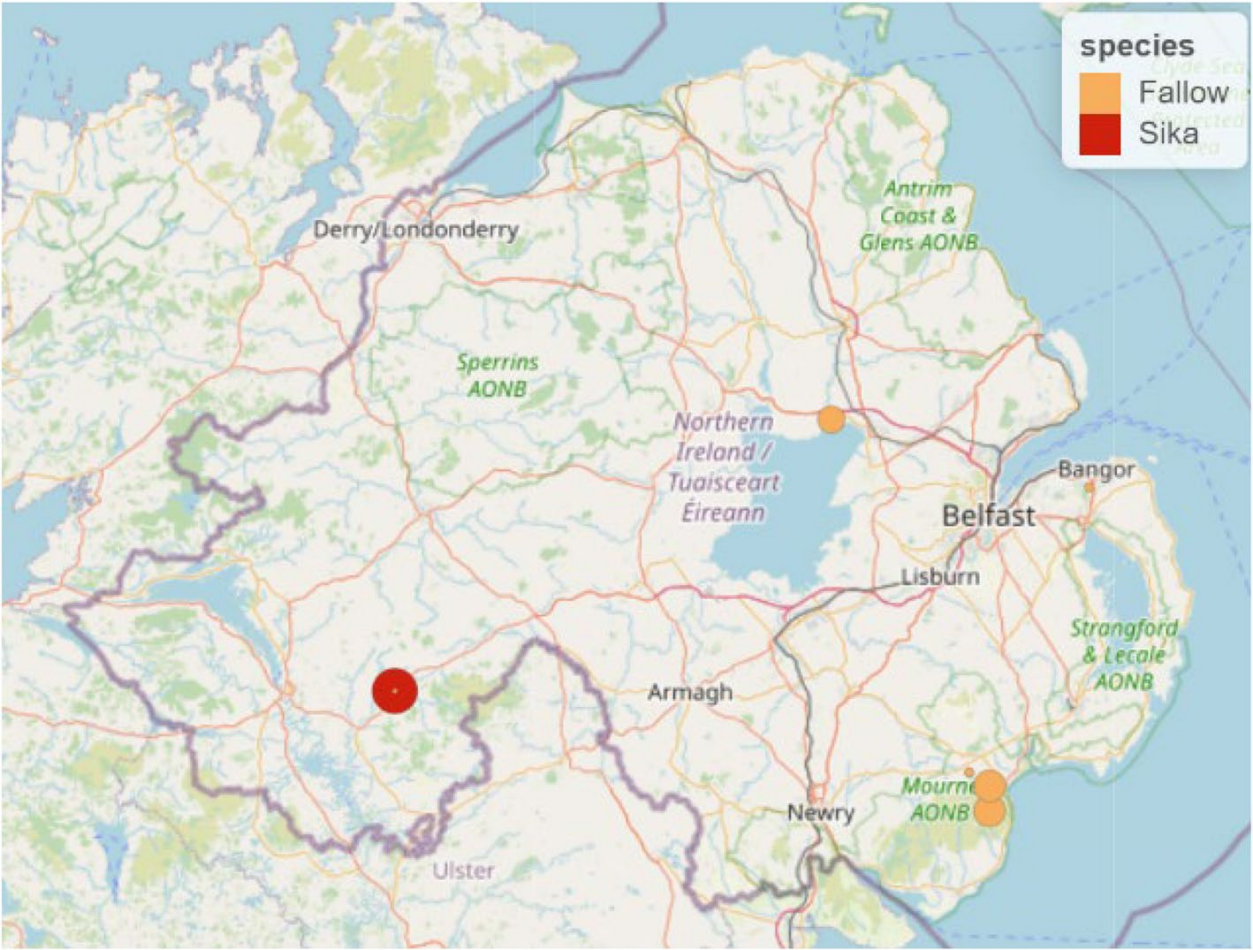
Sample maps of hunter-harvested blood samples collected from September 2022-March 2023, by species and weighted by area (*n* = 116)

Blood samples from the jugular vein of freshly culled deer were submitted to the Agri-Food and Biosciences Institute’s Veterinary Surveillance Division (AFBI VSD) by trained hunters (= stalkers) at larder sites in Co. Fermanagh and Co. Armagh in the open season from September 2022 to March 2023 (*n* = 116). Sampling was opportunistic and stalkers were provided with sampling kits which contained individually numbered sterile vacutainers, sampling instructions and a submission form, to detail animal metadata (age, sex, species, date of cull and cull coordinates). The larder (a central site used to process carcases) in Co. Fermanagh is on the premises of a 1000-acre estate which arranges culling on site and over around 7000 acres of surrounding area, in the west of the country. The larder in Co. Armagh is managed by the Northern Ireland Forest Service (Department of Environment, Agriculture and Rural Affairs, DAERA) and receives carcasses from woodlands in the east of the country. Figure 1 indicates the culling sites reported by stalkers. Blood samples were collected on the day of culling, in sterile vials and refrigerated at + 4⁰C. Mean time from collection to processing was 3.9 days. On receipt, bloods were centrifuged at 10,000 g for 15 min, serum removed and stored at −20⁰C until tested. Samples were taken from 116 deer and a breakdown of location, sex and age is provided in Table 1.

**Table 1:** Breakdown of data for deer samples submitted to AFBI VSD September 2022 to March 2023 (*n* = 116)

	Species (*n* = 116)			Sex (*n* = 116)			Age Category^a^ (*n* = 116)			
	*Fallow*	*Sika*	*Unknown*	*Male*	*Female*	*Unknown*	*Immature*	*Young*	*Adult*	*Unknown*
Co. Down	46	-	-	14	32	-	-	44	1	1
Fermanagh	1	46	-	12	33	2	4	30	9	4
North Down	2	-	-	-	2	-	-	-	-	2
Randalstown	18	-	-	8	10	-	-	17	1	-
Unknown	-	-	3	-	-	3	-	-	-	3
Total	67	46	3	34	77	5	4	91	11	10

^a^Age Category: Immature = < 1 year, Young = yearling to 3 years, Adult = ≥ 4 years (estimated by dental examination at stalker inspection)

### BVDV/BDV p80 ELISA

Exposure to BVDV or BDV was evaluated by Enzyme-Linked Immunosorbent Assay (ELISA), using Applied Biosystems PrioCHECK™ Rum. BVD/BD p80 Ab Serum & Milk blocking ELISA Kit (ThermoFisher Scientific) (*n* = 116). Serum and controls were tested according to the manufacturer’s instructions. Antibody levels were expressed in terms of percentage inhibition (%INH) and interpreted as %INH < 50 = negative, 50 ≤ %INH < 80 = weak positive, %INH ≥ 80 = positive. Manufacturer’s analytical sensitivity is 97% and specificity is 98% for BVDV and 96% sensitivity and 100% specificity for BDV.

### Pestivirus Virus Neutralisation Assay

Initially, samples were heat inactivated at 56 °C for 30 min and diluted to 1:4 with maintenance media for each tissue culture cell line. Eagle's Minimal Essential Media for foetal calf lung cell line for BVDV, Glasgow Minimal Essential Medium BHK-21 for lamb kidney cell line for BDV (Gibco, ThermoFisher Scientific). Each serum sample was titrated, in duplicate, in a doubling dilution series from 1/8 to 1/8192 on a sterile dilution plate, with one column containing no-virus control. An equal volume of 100TCID50 of the relevant virus, BVDV (BVDV-1a, field isolate, accession no. PP374763) or BDV (Moredun BDV-1a isolate, APHA, accession no. U65023.1) was added to each serum dilution in the series and incubated at 37 °C for 30 min, in the presence of 5% CO2. From this plate, serum/virus (100µL) was transferred to monolayered plates containing the appropriate cell culture along with cell-only, serum-only and virus controls (100TCID_50_, 10TCID_50_, 1TCID_50_, 0.1TCID_50_) and incubated at 37 °C, 5% CO2, for 4–6 days and assessed daily cytopathogenic effect (CPE) in the cells. Cells were then fixed by addition of 10% formalin (50 μl) per well and incubated at 37 °C for 30 min. Endogenous peroxidases were neutralised by the addition of 100 μl of 1% H2O2 per well, incubated at 37 °C for 5 min. Viruses were visualised and titres recorded by the addition of *pestivirus* monoclonal antibody (WB103/105, APHA), goat anti-mouse peroxidase (Jackson Immunoresearch Labs Inc, PA) and diamino benzidine (DAKO) chromogenic substrate, under the light microscope. Cut-off of viral neutralisation at sample titres of ≥ 1/8 was applied and a lower than fourfold difference between viral titres was taken as inconclusive for differentiation, as per the World Organisation for Animal Health (WOAH) guidelines [44].

### IBR (BoHV-1) gB Blocking ELISA

IDEXX IBR gB X3 (IDEXX Switzerland GmbH, Switzerland) ELISA was used according to the manufacturer’s instructions (*n* = 116). Antibody levels were expressed as a blocking percentage and interpreted as Blocking % < 45 Negative, 45 ≤ Blocking % < 55 Suspect, Blocking % ≥ 55 Positive.

### Figures

Maps were produced in R Studio using mapview package [45].

## Results

**Fig. 2:**
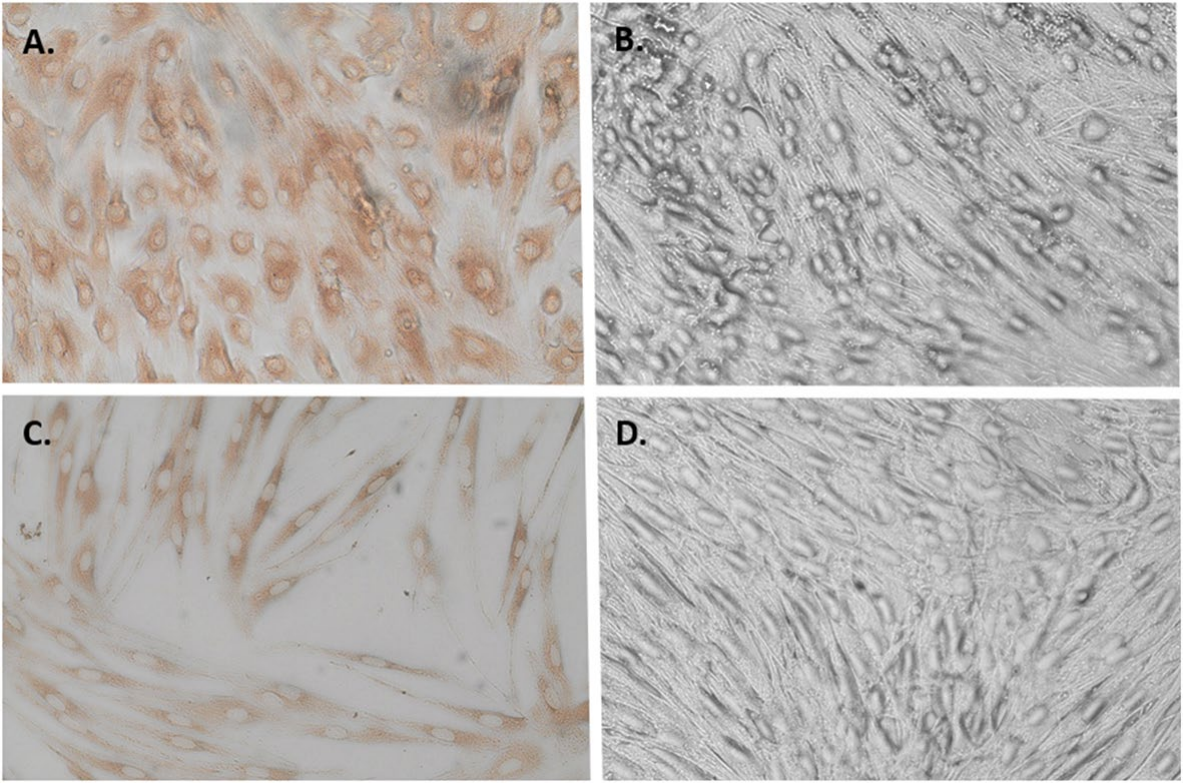
Virus Neutralisation Test images per cell line. BVD and BD viruses were introduced to the cells in the presence serum pestivirus antibodies isolated in wild sika deer. Immunoperoxidase was used to visualise virus inside the cells on light microscope at × 200 magnification (Olympus Lifesciences, MA). Images were captured using the Olympus DP28 Digital camera. **A** Foetal Calf Lung (FCL) cells. Pestivirus antibodies failed to neutralise the BVDV-1a field isolated strain at titre of 1/128. **B** Pestivirus antibody has neutralised the BVDV in the FCL cells at titres of 1/64. **C** Lamb Kidney (LK) cells. Pestivirus antibodies failed to neutralise the Moredun BDV-1a isolated strain at titre of 1/256. **D** Pestivirus antibody has neutralised the BDV in the LK cells at titres of 1/128

On BVDV/BDV p80 ELISA, 3/116 samples (2.6%) were positive for *pestivirus* (%INH 76.86 (low positive), 84.13 and 92.80 (positive), all from sika deer. On VNT of these positive samples, virus neutralisation titres were indistinguishable for BVDV/BDV in two samples (1/64 titre each on sample 22–54 and 1/128 titre each on sample 23–59) and two-fold higher for BDV than BVDV in the remaining samples, precluding viral differentiation. Figure 2 demonstrates VNT viral antibody titre determination in each cell line. Viral neutralisation titre and %INH levels were positively corresponding in each case (Table 2). On IBR (BoHV-1) gB ELISA, 0/116 samples (0%) were positive for BoHV-1 (%Blocking value range −138.66 to 24.76).

**Table 2:** Virus neutralisation titres against Bovine Viral Diarrhoea Virus (BVDV-1a/*Pestivirus A*) and Border Disease Virus (BDV/*Pestivirus D*) and ELISA % inhibition results for each deer samples with an antibody response to the *pestivirus* p80 antibody

Sample ID	BVDV-1a VNT Titre	BDV VNT Titre	ELISA %INH
22–54	1/64	1/64	84.13
22–55	1/16	1/32	76.86
23–59	1/128	1/128	92.80

## Discussion

The results presented here indicate a low seroprevalence of antibody to *pestivirus* (2.6%) and an absence of antibody to BoHV-1 in the sampled deer.

The *pestivirus* seroprevalence in the present study is comparable to many other studies, in a variety of deer species globally. Similar limitations of cross-reactivity and inconclusive speciation on ELISA and VNT were noted elsewhere [35, 46, 47]. European studies report anti-*pestivirus* antibodies in red deer with prevalences ranging from 0.4% in Great Britain [35] to 19.5% in Spain [20], with prevalence mostly ≤ 6.0%. The only all-Ireland study detected anti-*pestivirus* antibody in 1.5% of fallow and red deer, with positive samples from the Republic of Ireland only [34]. The highest European *pestivirus* seroprevalences in deer were found in reindeer at 32.0% [47], with the highest in roe of 12.3% [48], red 5.9% [46] and fallow 1.2% [17]. Prevalence varied widely across the rest of the world, with most (20/28) studies from North America. All anti-*pestivirus* antibody detected in the present study originated from sika deer samples. Although no European studies have previously identified *pestivirus* species in sika deer, seroprevalence in Japan has been reported at 3.0% [49]. Although it was not possible to distinguish between BVDV and BDV exposure in the present study, previous studies indicate that BDV seroprevalence in free-living deer in Europe is low, e.g., 0.3% was reported in Germany [17] and 0.1% in Spain [50]. While little is known about the impact of this virus on deer, it has been shown to cause significant disease and high mortality in other wild ungulates such as mouflon and chamois [51–53].

**Fig. 3:**
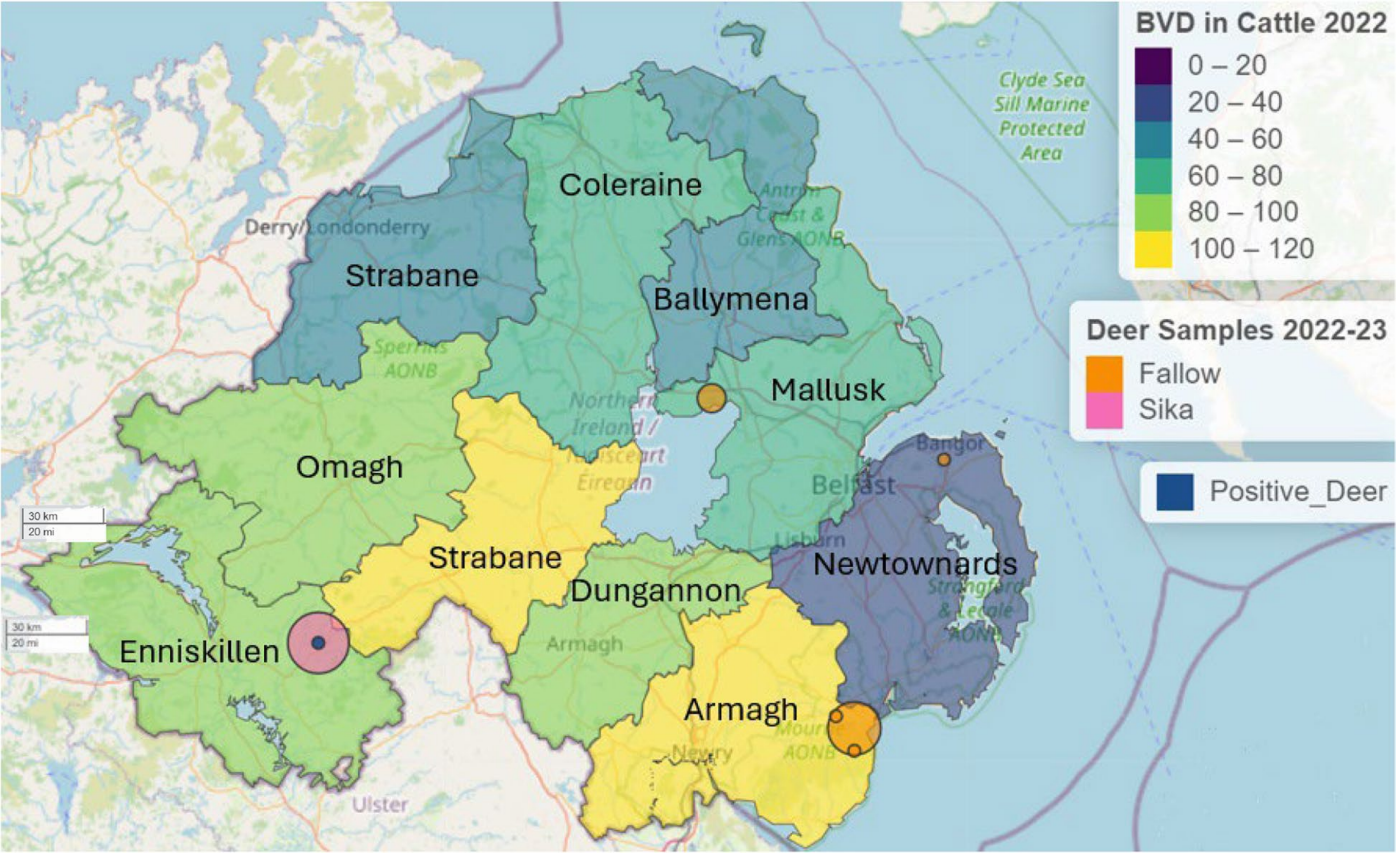
Choropleth map showing all reported BVD positive cattle in Northern Ireland in 2022 (*n* = 778, min = 36, max = 120) overlayed with weighted points for deer samples collected for the present study (*n* = 116, min = 3, max = 47) and samples positive for pestivirus on p80 ELISA (*n* = 3). Cattle data supplied by Animal Health and Welfare Northern Ireland

All *pestivirus* positive samples in this study originated from a larder in Co. Fermanagh. Sampling in this area accounted for 39.5% of the overall submissions in the study. County Fermanagh is in the south-west of Northern Ireland, which has some of the highest recorded seroprevalence of BVDV in cattle (Fig. 3), and borders counties of the Republic of Ireland with relatively high deer densities [54]. It is of note that all *pestivirus* seropositive deer were sika. While this could indicate a species-specific susceptibility to the pathogen, this is not supported by other studies. Instead, the logistics of sampling in this study led to a bias toward sika deer collected in this region, in which other factors including those mentioned above are likely to drive higher disease exposure, and further studies using a more representative population would be recommended.

Few studies have explored BoHV-1 in deer species, especially in the main wild European species: red, roe and fallow. Two European studies have reported seroprevalence in red deer of 23.0% and 25.5%, roe 10.5% and 1.7%, and fallow 2.2% and 23.1%, respectively [32, 55]. An all-Ireland multi-pathogen survey found low overall seroprevalence of 1.5%, and none from samples collected in Northern Ireland, in agreement with the present study. In the Irish study, the majority of positive animals (4/7) were fallow, followed by one each of red, sika and an unknown species [34]. Experimental in vivo infection and transmission studies are lacking for BoHV-1 in deer, particularly in regard to the mode of transmission, virulence and establishment of latent infection in deer.

BDV neutralising antibody titres of ≥ 1/8 were demonstrated in all samples, and were greater than anti-BVDV titres in one sample; however, the threshold for differentiation was not met. Failure to differentiate between these *pestiviruses* could imply cross-reactivity between strains, response to a *pestivirus* not represented in this panel, response to an atypical *pestivirus* strain, such as Pestivirus H (Hobi-like virus), or previous infection of these animals with both *pestivirus* strains (BVDV and BDV). Similarities between diagnostic targets of *pestiviruses* can complicate analyses and could have implications for eradication policies, with cross-reactivity and cross-neutralisation seen between BVDV, BDV and a *pestivirus* associated with porcines, Classical Swine Fever Virus (CSFV, *Pestivirus C*), especially on antibody-response related tests [56–58]. The VNT is considered the World Office for Animal Health (WOAH) gold standard test for *pestiviruses* [44], and thus would be recommended for inclusion in post-eradication surveillance in both livestock and wildlife where positive samples are indicated by other methods.

It is important to understand the epidemiology of endemic livestock-associated pathogens in the deer population as potential maintenance hosts of pathogens, with possible impacts on eradication and control programmes. As livestock disease control initiatives gain success, the relative importance of extrinsic re-exposure risk increases [35]. Pathogen maintenance in wildlife alongside an increase in disease naïve livestock, i.e. a newly susceptible population, may therefore hamper eradication efforts [14, 59]. The present results suggest that these concerns are allayed for BVDV and BoHV-1 in deer, but it would be prudent to continually monitor wildlife during and following virus eradication in livestock.

## Limitations

As with many wildlife studies, the unavailability of species-specific tests and species cells for VNT, necessitated the use of non-specific materials. The BVDV/BDV kit detects antibodies raised to the conserved p80 protein and the IBR gB kit is a competitive ELISA. These kits and the FCL and LK cells used in this study have been used in off-target species elsewhere [14, 42, 55, 60]. Samples for this study could only be legally obtained through hunting during autumn and winter months, possibly causing a temporal bias. While VNT results concurred with those of serology, the sample size was low and conducting VNT on seronegative samples would have allowed fuller investigation of the consistency between VNT and ELISA results; however, resources did not allow this to be pursued. Other limitations to this study include a small and opportunistically selected sample population, although a lack of real estimate numbers for wild deer in the province makes it difficult to assess to what degree bias exists in this respect. Sample bias by both species and provenance is possible: collection was heavily skewed to the southwest of the country with a clear species population divide by area. Greater population diversity and a structured sample would be recommended for future studies.

## Conclusion

The present results indicate that the presence of BVDV/BDV and BoHV-1 in wild deer is unlikely to significantly impede control efforts for these pathogens in Northern Irish livestock. Continued surveillance, however, is recommended. Viral species differentiation, by VNT or genetic sequencing is also advised in future work, to prevent inaccurate reporting, which could jeopardise post-eradication disease freedom status.

## Data Availability

The datasets supporting the conclusions of this article are included within the article will be made available on request.

## Abbreviations

AHI: Animal Health Ireland
BD: Border Disease
BDV: Border Disease Virus
BoHV: Bovine Herpes Virus
BVD: Bovine Viral Diarrhoea
BVDV: Bovine Viral Diarrhoea Virus
CPE: Cytopathogenic Effect
ELISA: Enzyme-linked Immunosorbent Assay
IBR: Infectious Bovine Rhinotracheitis
PI: Persistently Infected
ROI: Republic of Ireland
TI: Transiently Infected
VNT: Virus Neutralisation Test
WOAH: World Office for Animal Health
%INH: Percent Inhibition
